# Predictive prosthetic socket design: part 1—population-based evaluation of transtibial prosthetic sockets by FEA-driven surrogate modelling

**DOI:** 10.1007/s10237-019-01195-5

**Published:** 2019-06-29

**Authors:** J. W. Steer, P. R. Worsley, M. Browne, A. S. Dickinson

**Affiliations:** 1 Bioengineering Science Research Group, Faculty of Engineering and Physical Sciences, University of Southampton, Southampton, UK; 2 Clinical Academic Facility, Faculty of Health Sciences, University of Southampton, Southampton, UK

**Keywords:** Finite element analysis, Amputation, Statistical shape modelling, Principal component analysis

## Abstract

It has been proposed that finite element analysis can complement clinical decision making for the appropriate design and manufacture of prosthetic sockets for amputees. However, clinical translation has not been achieved, in part due to lengthy solver times and the complexity involved in model development. In this study, a parametric model was created, informed by variation in (i) population-driven residuum shape morphology, (ii) soft tissue compliance and (iii) prosthetic socket design. A Kriging surrogate model was fitted to the response of the analyses across the design space enabling prediction for new residual limb morphologies and socket designs. It was predicted that morphological variability and prosthetic socket design had a substantial effect on socket-limb interfacial pressure and shear conditions as well as sub-dermal soft tissue strains. These relationships were investigated with a higher resolution of anatomical, surgical and design variability than previously reported, with a reduction in computational expense of six orders of magnitude. This enabled real-time predictions (1.6 ms) with error vs the analytical solutions of < 4 kPa in pressure at residuum tip, and < 3% in soft tissue strain. As such, this framework represents a substantial step towards implementation of finite element analysis in the prosthetics clinic.

## Introduction

The prosthetic socket provides the critical attachment between the residual limb following amputation and the prosthetic device. Each socket is bespoke to the user and is designed in a manual and iterative process by a prosthetist. This process is dependent on their skill and experience, as well as patient feedback (Paterno et al. 2018) with no quantitative prediction of fit prior to the manufacture of the socket. As a result, on average nine fitting and adjustment sessions are required in the first year following amputation (Pezzin et al. 2004). Inadequate socket fit leads to pain and potentially device rejection, restricting activities of daily living (Hsu and Cohen 2013). To ensure a good socket fit, clinicians perform a series of geometrical modifications to the captured shape of the individual’s residual limb, known as rectification, targeting optimal load transfer. Traditionally, this involved physical modification of a positive plaster mould. However, digital technologies are becoming more prevalent within the clinical community (Whiteside et al. 2007; Karakoç et al. 2017). Commonly, this approach involves using a surface scanner to digitise the limb’s surface shape, performing the patient-specific rectifications in a CAD environment and manufacturing a mould to form the socket within a central fabrication facility (Saunders et al. 1985; Oberg et al. 1989; Sanders et al. 2007).

The residual limb after lower limb amputation is created by forming a soft tissue pad over the resected bone (Smith and Fergason 1999). The complex device/patient geometry together with the significant differences in biological and prosthetic material properties creates a challenging environment for appropriate load transfer. The skin at the interface is subject to high pressure and shear gradients, which frequently lead to discomfort (Lyon et al. 2000) and potentially the formation of chronic wounds, termed pressure ulcers or stump ulcers (Yusuf et al. 2015). This effect is exacerbated by elevated temperature and humidity (Hachisuka et al. 2001; Kottner et al. 2018) which lower the skin’s tolerance to load, in addition to diurnal fluctuation in residual limb volume (Zachariah et al. 2004). Further, sustained sub-dermal soft tissue strains can lead to deep tissue injury (DTI) (Portnoy et al. 2009a; Loerakker et al. 2011; Oomens et al. 2015), which may require further amputation surgery (Highsmith et al. 2016).

There has been considerable research into using biomechanical metrics as surrogates for the goodness-of-fit of the prosthetic socket, in particular interface pressure and shear. This has either been measured with interface sensors (Goh et al. 2004; Dou et al. 2006; Dumbleton et al. 2009; Tang et al. 2017) or predicted using finite element analysis (FEA) (Jia et al. 2005; Dickinson et al. 2017). FEA has been identified as a potential tool to assist the prosthetist in their design process, by providing a prediction of fit prior to manufacture (Zhang et al. 1998). However, there are substantial barriers to clinical implementation of these techniques including difficulty in obtaining imaging data, lengthy solver times for the models and the need for a trained user to develop and interpret the FE model (Dickinson et al. 2017). Further, despite the first FE model of a lower limb amputee being published in 1988 (Reynolds 1988), research in this field has not advanced at the rate of many implanted prosthetic devices where tools to simulate the variation in performance across a population are well established (Bryan et al. 2010; Taylor and Prendergast 2015; Ragkousis et al. 2016) or in the prediction of sub-dermal soft tissue strains during seating (Al-Dirini et al. 2016; Luboz et al. 2017). This provides the motivation and objective for the present study, which aimed to develop a surrogate model to allow equivalent predictions to single FEA solutions, across a broad population of anatomical, surgical and design variability, with sufficiently reduced computational expense for clinical use.

## Methods

### Baseline FE model

**Fig. 1:**
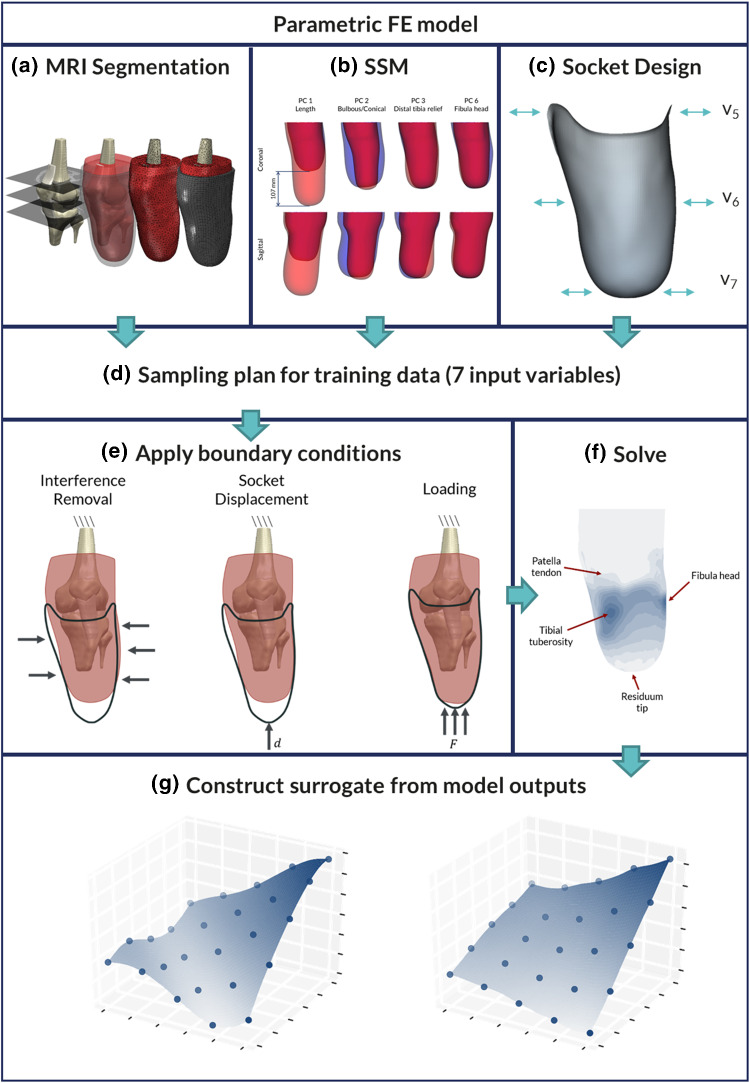
Flowchart of the developed workflow. **a** Segmentation of the MRI scan and creation of the FE mesh, **b** SSM from PCA of 30 surface scans, **c** parametric model of TSB socket design, showing the three design variables used to control the press fit at the proximal, mid and distal regions, **d** Latin hypercube sampling plan of the seven input variables to the parametric model, **e** application of model boundary conditions including the socket donning and loading, **f** solution of the FE models as training data, highlighting the regions of interest across the limb, **g** creation of the surrogate model based on the FE simulations. Dots denote the training data, and the surface shows the fitted function

The baseline FE model was generated from a single MRI scan of a unilateral transtibial residual limb (MAGNETOM Spectra, Siemens Healthcare GmbH, Germany; 3.0 mm slice thickness, 0.5 mm in-slice pixel size, T1-weighted), who provided informed written consent (Fraunhofer IPA #2016_BLM_0009), obtained with secondary data ethical approval (ERGO#29927). The bones, a simplified cartilage-meniscus structure, and the patellar tendon were segmented (ScanIP N-2018.03, Synopsys Inc., USA, Fig. 1a), and other soft tissues were treated as a single body. The meniscus layer was used to facilitate load transfer between the tibia and femur, although sliding was not permitted. The FE mesh was generated with 40272 quadratic tetrahedral elements and imported into an FEA solver (ABAQUS 6.14, Dassault Systèmes, Vèlizy-Villacoublay, France). A segmented prosthetic liner was meshed around the residuum with 5882 structured hexahedral elements. Subsequently, a baseline socket shape was extracted from the external surface of the liner, representing a total surface bearing design (TSB), meshed with 1851 quadrilateral elements. The limb–liner interface was tied, and a Coulomb friction model with coefficient of friction of 0.5 was defined at the liner–socket interface (Cagle et al. 2018).

Socket donning was simulated under displacement control, to generate initial interference pressure and shear between the limb and the socket, from an initial distance of 20 mm. Following donning, a 400 N axial load at the base of the socket (representative of standing) was applied (Fig. 1e). The proximal cut surfaces of the femur and tendon were constrained in all degrees of freedom. The model was solved using implicit analysis, and all loading conditions were static.

### Residuum shape population model

A statistical shape model (SSM) was used to introduce population-representative morphological variation into the FE model. SSM has previously been used extensively to characterise shape variation in biological tissues across an anatomical population (Barratt et al. 2008; Bryan et al. 2010; Woods et al. 2017).

For the present study, 30 surface scans of anonymised rectified transtibial plaster casts were used to generate a principal component analysis (PCA) model. These surface scans were aligned and registered to the external surface mesh of the limb extracted from the MRI scan according to a previously verified methodology using the open-source AmpScan package (Dickinson et al. 2016). The 30 aligned and registered scans, as well as the mesh extracted from the MRI scan, were used as input data for the PCA model. The PCA model was developed using singular value decomposition on a mean centred dataset of mesh vertex locations (Galloway et al. 2012).

**Fig. 2:**
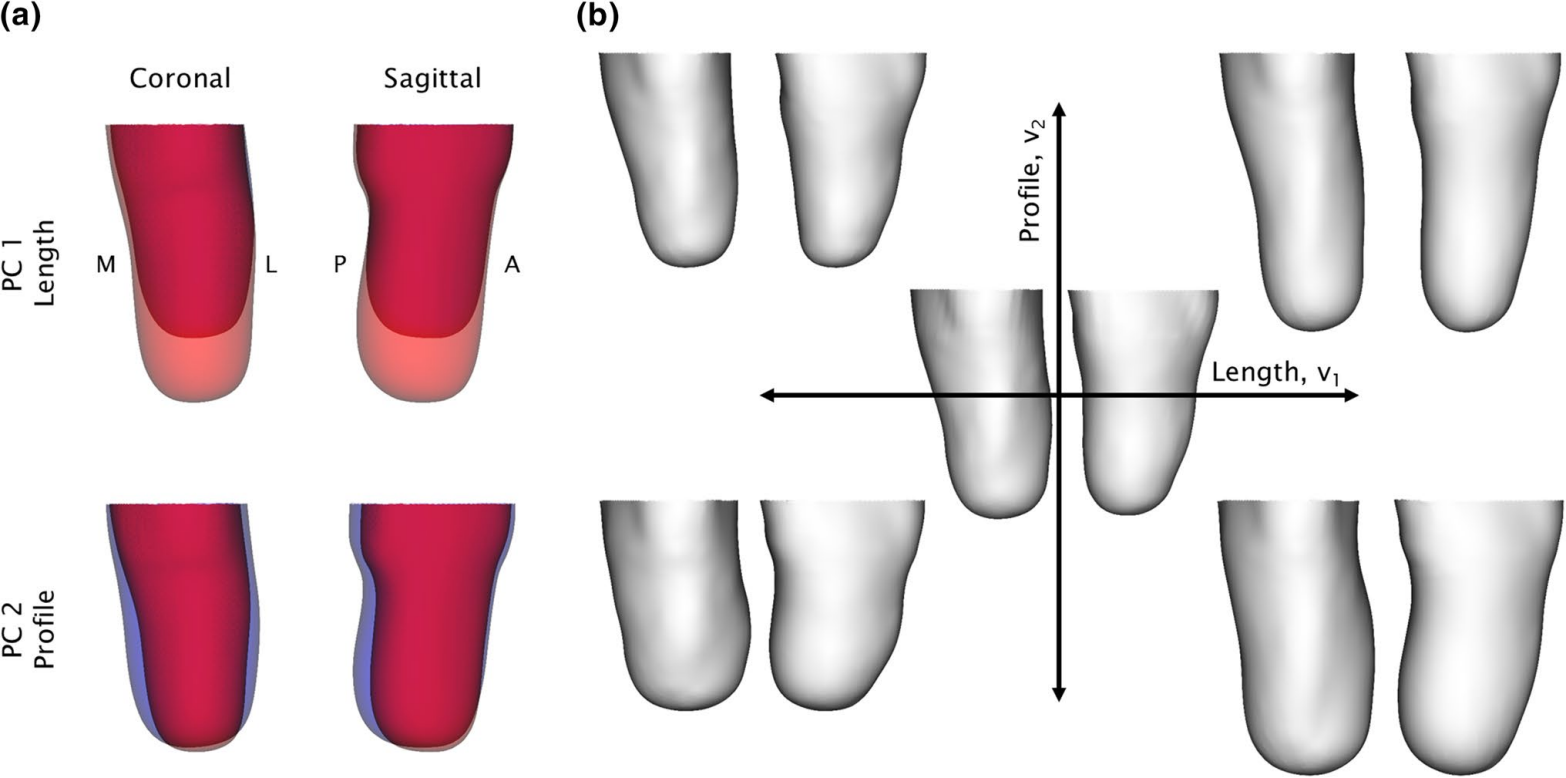
Results of the SSM. **a** Effect of varying the weights of PCs 1 and 2 by ± 1 *σ* with respect to the mean shape in the coronal plane, with the medial and lateral aspects labelled M & L, and the sagittal plane, with anterior and posterior aspects labelled A & P. **b** Effect of varying the baseline mesh from the MRI scan with the first two PCs, while fixing the remaining PCs

The first two PCs of the SSM (Fig. 2a) were found to be dominated by residuum length (i.e. the surgical amputation height) and profile (i.e. how conical or bulbous the limb is) which represented 91% of the population variance (83% PC1, 8% PC2). These two PCs were selected to introduce surgical and anatomical variation into the FE model, respectively. Higher PCs were neglected as they included socket rectification features which were not relevant to this study. For the parametric FE model, the weights of PCs 1 and 2 were constrained within the range of ± 1 standard deviation, *σ*, about the population mean, while the weights of PC 3 onwards were fixed at the original values of the MRI scan’s baseline mesh shape (Fig. 2b).

### Parametric FE model

The FE model was parameterised using seven input variables (Table 1). Four represented morphological variability of the residuum, and three represented the prosthetic socket design.

**Table 1:** Parametric FEA input variable name and bounds for the seven variables in the parametric FE model

Input variable name	Lower bound	Upper bound
PC 1 (residuum length), $v_{1}$	− 1 *σ* (short)	+ 1 *σ* (long)
PC 2 (residuum profile), $v_{2}$	− 1 *σ* (bulbous)	+ 1 *σ* (conical)
Bone length, $v_{3}$	− 15%	+ 30%
Soft tissue stiffness (*E*, kPa), $v_{4}$	35	55
Proximal press fit, $v_{5}$	− 2%	+ 6%
Mid press fit, $v_{6}$	− 2%	+ 6%
Distal press fit, $v_{7}$	− 2%	+ 6%

The four morphological variability parameters were defined using the two statistical shape model PCs described above, soft tissue stiffness and the tibia length relative to the residuum length, where 0% represented the same relative length as the baseline model. The soft tissue stiffness was defined using a linear range of elastic modulus values between 35 and 55 kPa, with the Poisson’s ratio fixed at 0.49. These bounds were selected to cover the range between stiff, flaccid muscle and contracted muscle (Portnoy et al. 2009b). This stiffness was converted to an equivalent neo-Hookean material to model the nonlinear behaviour of soft tissue (Palevski et al. 2006):

1 $$C_{1} = \frac{E}{{4\left( {1 + v} \right)}}$$

2 $$D_{1} = \frac{{6\left( {1 - 2v} \right)}}{E}$$

where $E$ and $v$ are the elastic modulus and Poisson’s ratio and $C_{1}$ and $D_{1}$ are the constitutive parameters of the slightly-compressible neo-Hookean strain energy density function, $W$, given by:

3 $$W = C_{1} \left( {I_{1} - 3} \right) + \frac{1}{{D_{1} }}\left( {J - 1} \right)^{2}$$

where $I_{1}$ is the deviatoric strain invariant defined as $\overline{I_{1}} = \overline{\lambda_{1}}^{2} + \overline{\lambda_{2}}^{2} + \overline{\lambda_{3}}^{2}$, the deviatoric stretches are given by $\overline{\lambda_{i}} = J^{-1/3} \lambda_{i}$, and J is the total volume ratio. The prosthetic liner was also modelled as a hyperelastic material, while the bones, tendon and socket were all modelled as linear elastic (Table 2).

**Table 2:** Material properties applied to the modelled structures

Structure	*E* (MPa)	$v$	*C* (kPa)	*D* (MPa^−1^)	References
Bone	12,000	0.3	–	–	Reilly and Burstein (1975)
Tendon	400	0.49	–	–	Stäubli et al. (1999)
Meniscus	59	0.49	–	–	Pena et al. (2006)
Liner	–	–	37.6	0.54	Sanders et al. (2004)
Socket	1500	0.3	–	–	Lee et al. (2004)

Three variables were used to define the shape of the socket, and represented the ‘press fit’ at proximal, mid and distal portions of the socket between − 2% and + 6%, defined as a percentage reduction in the radial distance of each node to the first principal axis of the tibia, calculated from the first PC of the mesh nodes (Fig. 1c). These design variables represented a simplified, parametric model of a TSB socket (Fernie and Holliday 1982; Staats and Lundt 1987).

### Volumetric mesh morphing

**Fig. 3:**
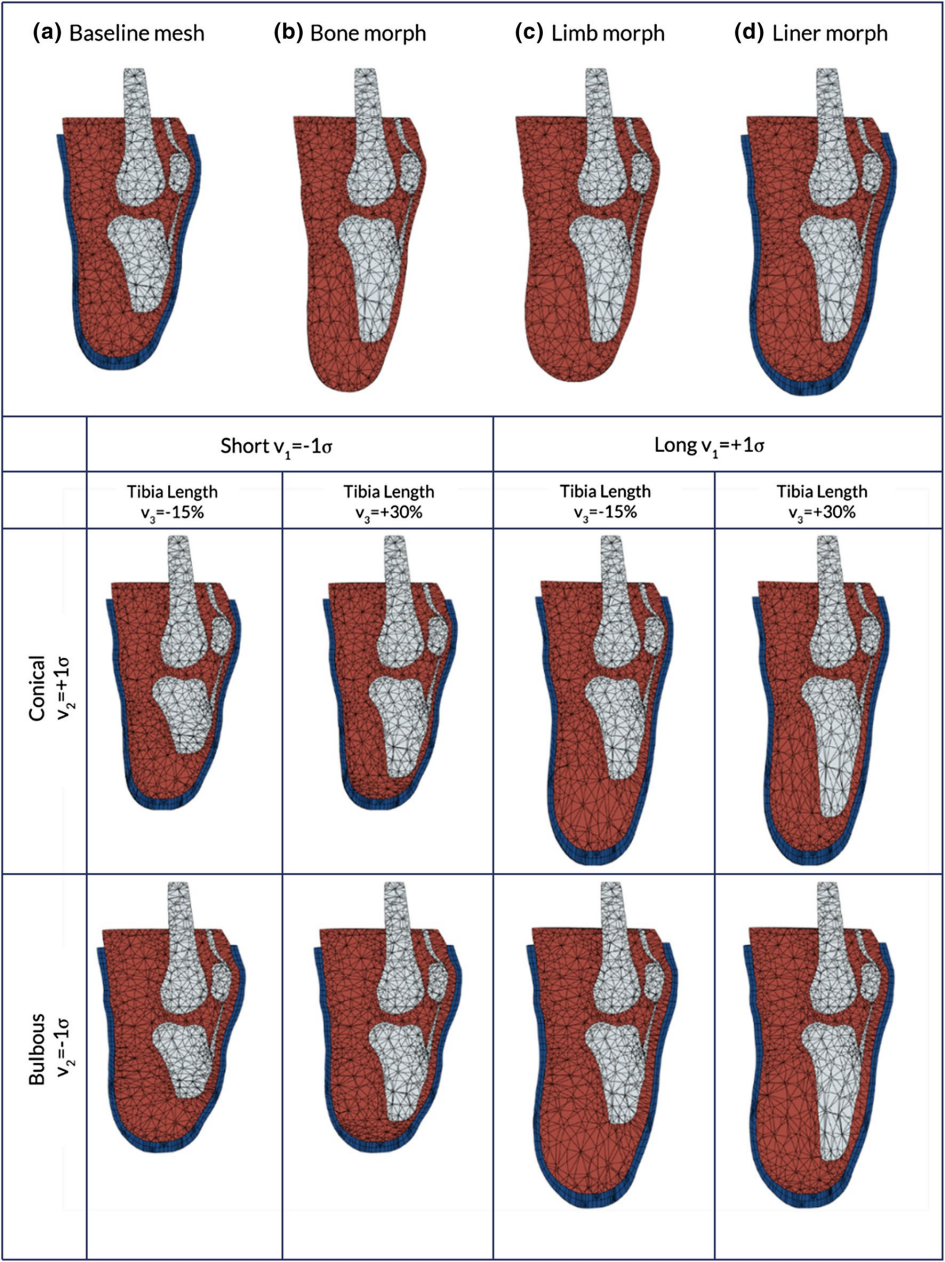
Procedure used for morphing the baseline FE mesh (**a**), through modifying the bone length (**b**), then morphing the external shape of the limb to match the SSM (**c**) and finally morphing the external liner (**d**). The result of morphing the FE mesh to morphological parameters of the model informed by residuum length, v_1_, residuum profile, v_2_ and tibia length, v_3_. Soft tissue is displayed as red, bone as grey and the liner as blue. The mesh has been visualised using a planar cut that goes through the elements

Population variability was accounted for in the FE model by morphing the volumetric FEA mesh (Fig. 3). Mesh morphing was performed using radial basis functions (RBFs) based on the technique proposed by Forti and Rozza (2014). While a more comprehensive description can be found in their paper, a summary is detailed below.

The matrix containing mesh nodal coordinates ${\mathbf{X}}$ was morphed into their new coordinates ${\mathbf{Y}}$ by displacing a matrix of control points ${\mathbf{X}}_{\text{c}}$ to new coordinates ${\mathbf{Y}}_{\text{c}}$.

4 $${\mathbf{Y}} = \hat{f}\left( {\mathbf{X}} \right) = \varvec{c} + {\mathbf{QX}} + {\mathbf{w}}^{T}\varvec{\psi}\left( {\mathbf{X}} \right)$$

where ${\mathbf{w}}$ was a vector of the weights of the basis functions $\varvec{\psi}$, and vector $\varvec{c}$ and matrix ${\mathbf{Q}}$ were the parameters of the linear function included to express rigid translation/rotation.

The mapping function is defined between the initial position of the control points ${\mathbf{X}}_{c}$ and the final position ${\mathbf{Y}}_{c}$ using RBFs by evaluating $\psi \left( \left\|{{\mathbf{X}}_{{C_{i} }} - {\mathbf{X}}_{{C_{j} }} } \right\|\right)$, enabling the weights ${\mathbf{w}}$ and linear transformation terms $\varvec{c}$ and ${\mathbf{Q}}$ to be calculated by solving a set of linear equations. The mesh transformation is then defined by evaluating the RBFs at $\psi \left( \left\|{{\mathbf{X}}_{i} - {\mathbf{X}}_{{C_{j} }} } \right\|\right)$ and calculating ${\mathbf{Y}}$ from the pre-computed weights and linear transformation terms. Their method required no orthogonal projection or search algorithm and was not computationally intensive unless an extraneous number of control points was used.

A multi-quadratic biharmonic spline RBF was used, defined by $\psi \left( x \right) = \sqrt {x^{2} + r^{2} }$ where $r$ represents the scaling factor controlling the basis shape. To resolve the morphing of both the bone surface and the residual limb surface, the mesh morphing defined in Eq. (4) was performed in two steps. To morph the limb mesh $\varvec{X}$, two sets of control points were defined across the bony structures ${\mathbf{X}}_{{{\text{c,}}\,{\text{bone}}}}$ and the limb surface ${\mathbf{X}}_{{{\text{c,}}\,{\text{limb}}}}$, and the following procedure was used (Fig. 3):

1. ${\mathbf{X}}_{{{\text{c,}}\,{\text{bone}}}}$ was displaced to ${\mathbf{Y}}_{{{\text{c,}}\,{\text{bone}}}}$ to represent the new bone length and was used to morph $\varvec{X}$ and ${\mathbf{X}}_{{{\text{c,}}\,{\text{limb}}}}$ into their new locations (Fig. 3b)
2. The displacement field for ${\mathbf{X}}_{{{\text{c}},\,{\text{limb}}}}$ was defined by registering the control points onto the new limb surface from the SSM to generate the new locations ${\mathbf{Y}}_{{{\text{c}},\;{\text{limb}}}}$
3. ${\mathbf{X}}$ was then morphed a second time using ${\mathbf{Y}}_{{{\text{c}},\,{\text{limb}}}}$ into the final locations ${\mathbf{Y}}$ (Fig. 3c).

The meshes of the liner and socket were morphed based purely on the displacement field of the new limb surface from the SSM (Fig. 3d).

### Kriging surrogate model

Surrogate modelling enables fitting a continuous function to a set of training data across a multi-dimensional design space. New data points from the surrogate model can often solve several orders of magnitudes faster than expensive training data generation process, such as FE analyses. A full description and mathematical derivation of surrogate modelling, in particular Kriging-based models, can be found in Forrester et al. (2008).

The seven input variables were normalised into a unit hypercube. Latin hypercube sampling was used to generate the optimal distribution for the selected number of training data points (Morris and Mitchell 1995). A Kriging surrogate model was constructed from the outputs of the training data using the open-source pyKriging package (Paulson and Ragkousis 2015). The Kriging model was used over alternate RBFs due to its robust ability to model nonlinear behaviour, and enabled the expected error in the surrogate function to be calculated. A sensitivity analysis was performed between 25 and 200 points to determine the number of training data points required to accurately represent the input space (Table 2) for each of the model outputs, based on a test dataset of 75 points.

### FE model outputs

Pressure and shear at the liner–prosthetic socket interface were extracted from regions of interest (ROI) at the residuum tip, tibial tuberosity, fibula head and posterior calf (Jia et al. 2005, Fig. 1f). Sub-dermal soft tissue minimum principal strains were extracted around the soft tissues overlying the bony tibial prominence (Portnoy et al. 2009a). For all metrics, the 95th percentile magnitude was used across the values in the region of interest to quantify high values of pressure which cause socket discomfort while removing any mesh artefact stress peaks which may erroneously occur in the FE model. These metrics were used as the training data to construct the surrogate model.

### PCA-Kriging and real-time visualisation

In addition to localised predictions of biomechanical load at key regions over the residual limb from the surrogate model, a PCA-Kriging model was used to predict the full-field pressure and shear (Buljak 2010). Using the same formulation as the SSM, a PCA model was constructed from the training data pressure and shear values of all liner–socket interface nodes, named a statistical output model (SOM).

An individual surrogate model was constructed for each of the first 20 PCs from the SOM, which represented 99.9% of its variance. This approach was used instead of solving the surrogate on each node of the mesh, reducing the time to compute the Kriging models. This enabled a new full-field prediction, facilitating real-time visualisation of the model.

## Results

### Surrogate model

Numerical convergence was achieved for all the FEA simulations within approximately 30 mins per simulation (Intel Core i7-4790, 3.60 Ghz, 24 GB RAM). New data points from the ROI surrogate models were evaluated in 1.6 ms, representing an increase in solver speed of $\sim10^{6}$ times. The mesh morphing algorithm preserved quality throughout compared to the baseline mesh generated by ScanIP as demonstrated by the convergence of all the models. The mean and minimum Jacobian, which measure the deviation from the ideally shaped element were 0.56 ± 0.01 and 0.06 ± 0.02, respectively, across all meshes.

**Fig. 4:**
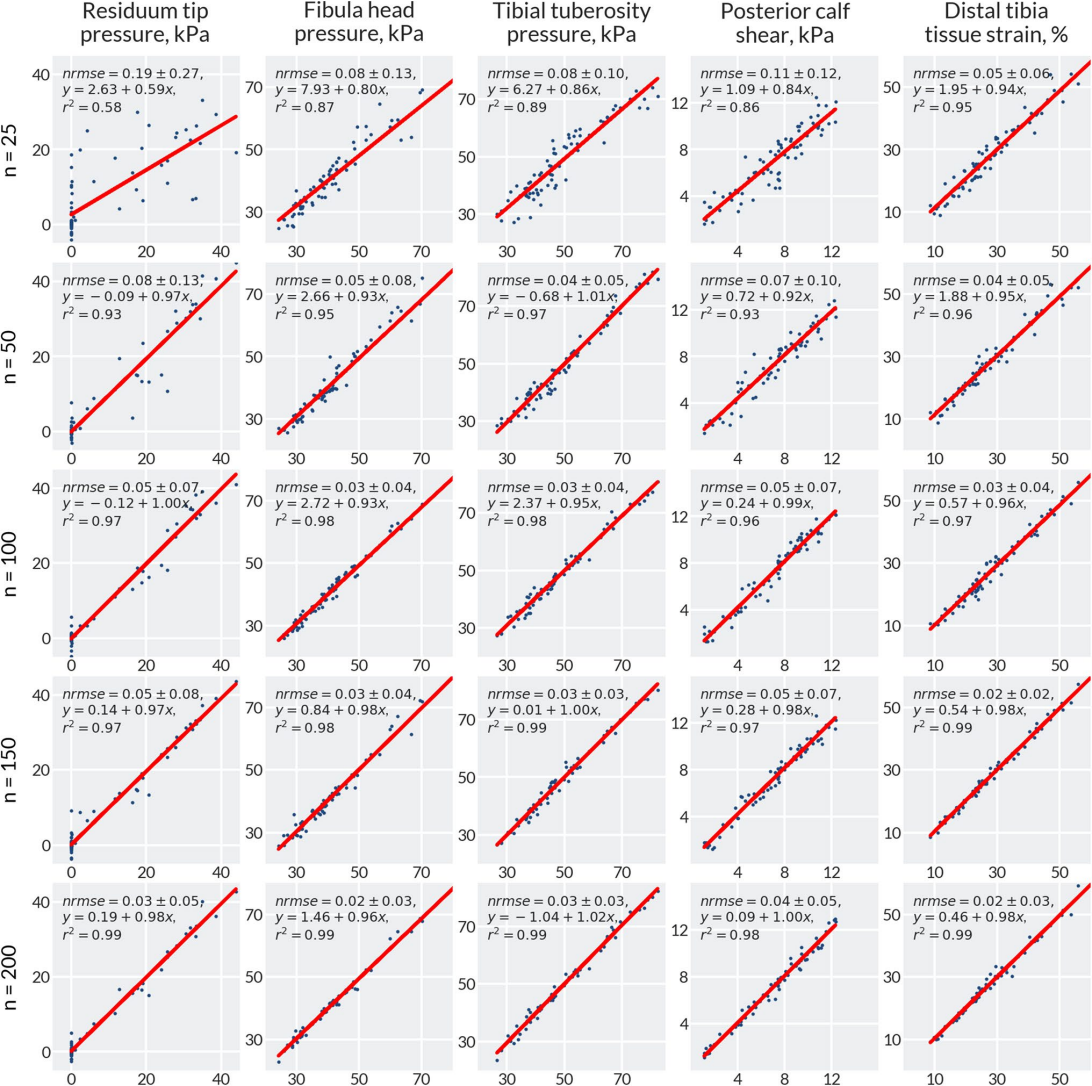
Regression analysis of the surrogate models with different numbers of training data points. In each plot, the *x*-axis gives 75 observed data points from the simulations and the *y*-axis gives the predictions from the surrogate model for the corresponding observed data points

Sensitivity analysis of the surrogate model demonstrated that the limb ROIs required different numbers of training data points (Fig. 4). The correlation between the training and observed data was very high, with $r^{2} > 0.9$ for all surrogate models apart from the 25 training data point model. However, analysis of the normalised root-mean-square error (NRMSE) demonstrated that there was still error in surrogate predictions. The highest error was observed at residuum tip, with an NRMSE of 8% for 50 training data points, falling to 4% for 150 data points. Further, the surrogate often predicted infeasible values of pressure less than 0 at the residuum tip due to difficulties in fitting a smooth function to the design space. The fibula head and tibial tuberosity pressure was predicted with an NRMSE of 4% for 50 data points.

The PCA-Kriging model enabled the output data to be reduced from 2977 to 20 data points. As such, only 20 surrogate models had to be computed and solved to predict the full-field output data. This facilitated real-time computation of the full-field pressure and shear data (44 ms). This was packaged into a custom graphical user interface to enable visualisation of the full-field data.

### Effects of anatomical variability

To investigate the effects of anatomical variability on the biomechanical response of the residual limb, the socket design press fit was fixed at + 1.0%. The residuum morphology was observed to affect the response at all the interrogated ROIs.

**Fig. 5:**
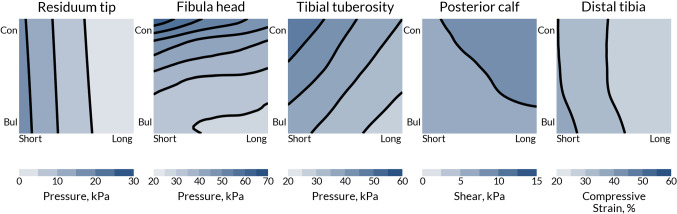
Interface pressure, shear and soft tissue strain at key ROIs of the model for fixed values of tissue modulus and tibia length ($v_{4} = 45 \,{\text{kPa}}, v_{5} = + 7.5\% )$, and a uniform + 1% press fit socket ($v_{5} = v_{6} = v_{7} = + 1.0$). The *x*-axis for each plot corresponds to residuum length, $v_{1}$, and the *y*-axis to residuum profile, $v_{2}$

Shorter residual limbs were predicted to generate higher residuum tip pressures and distal tibia soft tissue strains, as well as lower posterior calf shear (Fig. 5). Longer, more bulbous limbs were predicted to experience lower pressures over both the tibial tuberosity and fibula head.

**Fig. 6:**
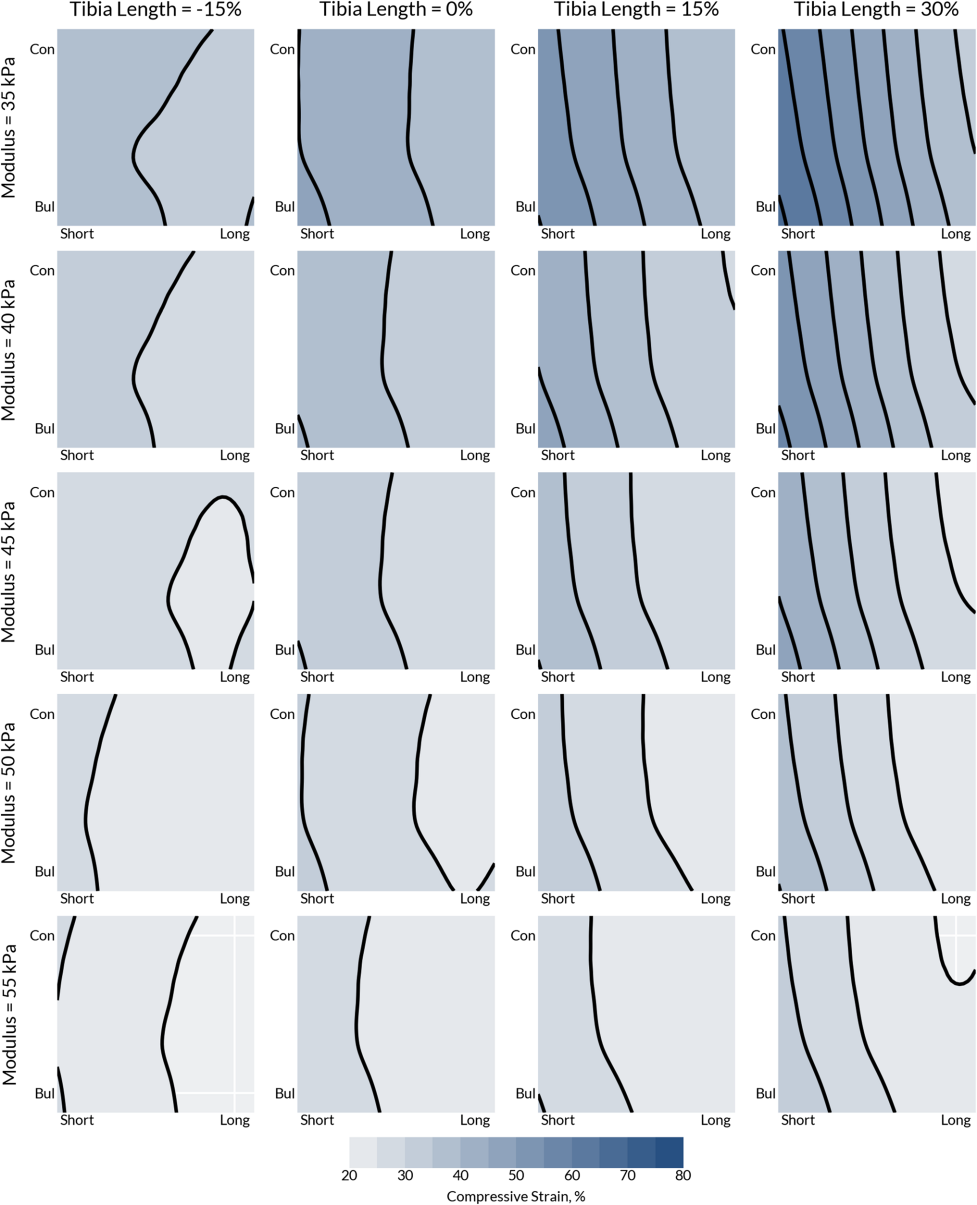
Distal soft tissue strain for different values of tibia length, $v_{3}$, and soft tissue modulus,$v_{4}$, for a + 1% press fit socket ($v_{5} = v_{6} = v_{7} = + 1.0$). The *x*-axis for each plot corresponds to residuum length, $v_{1}$, and the *y*-axis to residuum profile, $v_{2}$

The magnitude of soft tissue compressive strain and the soft tissue modulus was closely coupled, with the lower modulus resulting in substantially higher soft tissue strain (Fig. 6). Increasing the relative tibia length was also observed to generate higher soft tissue strain. Conversely, the tissue modulus only had a minor influence on the interfacial pressure and shear.

### Patient-specific socket design

**Fig. 7:**
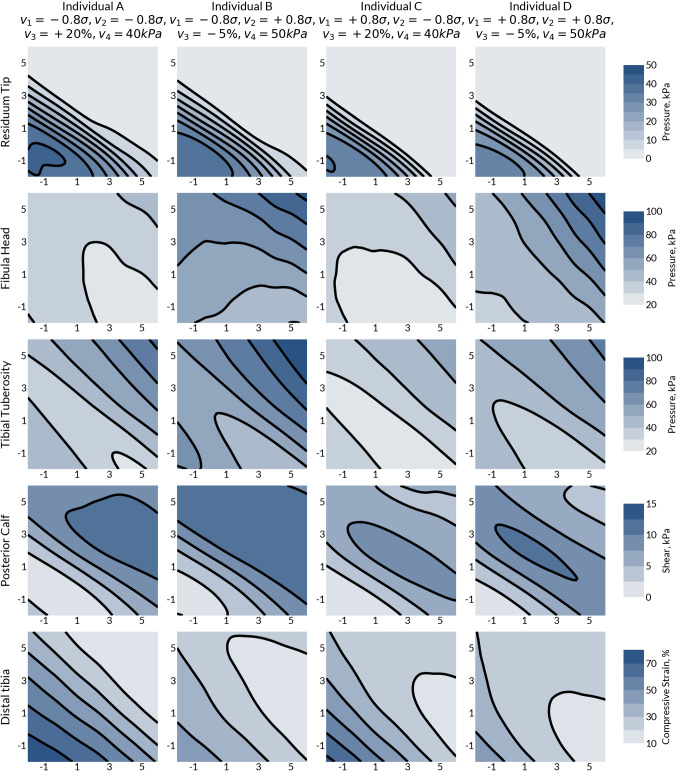
Effects of prosthetic socket design on the biomechanical response of the limb in each of the ROI. The *x*-axis of each represents the proximal press fit, $v_{5}$ in %, and the *y*-axis the distal press fit, $v_{7}$ in %. The mid press fit is the average of the proximal and distal press fit, $v_{6} = 0.5\left( {v_{5} + v_{7} } \right)$

**Fig. 8:**
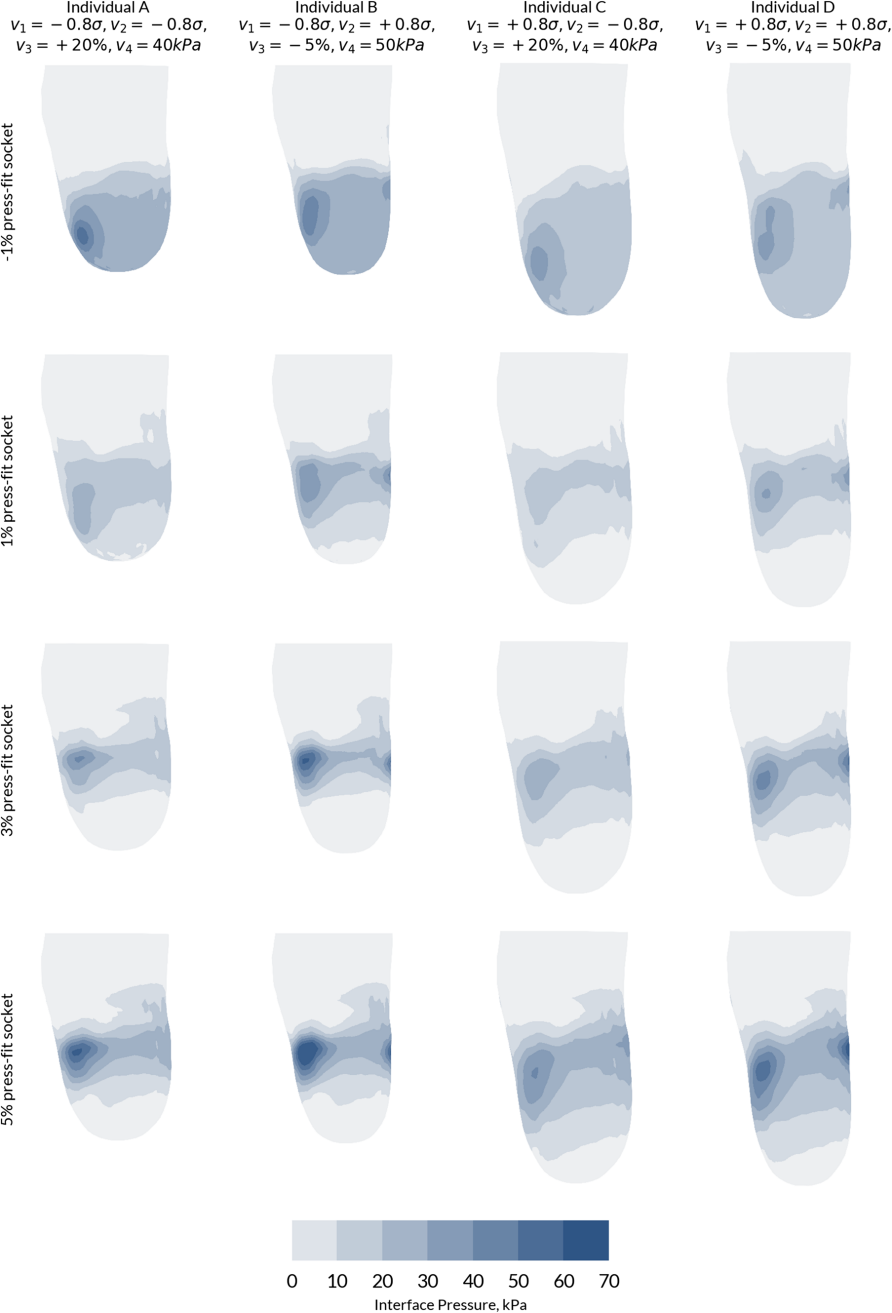
Interface pressure profiles for the four cases from the population with four different socket designs. Each press fit socket is designed so $v_{5} = v_{6} = v_{7}$. Four magnitudes of press fit corresponding to − 1, 1, 3 and 5% were selected. A $45^{ \circ }$ anterior-lateral view is presented, to visualise the pressure at the tibial tuberosity and fibula head

Case A represents a short, conical residuum with a long tibia and low tissue modulus (Figs. 7a, 8a); Case B is short and bulbous, with a short tibia and stiff soft tissue (Figs. 7b, 8b); Case C is long and conical, with a long tibia and low tissue modulus (Figs. 7c, 8c); Case D is long and bulbous, with a short tibia and high soft tissue modulus (Figs. 7d, 8d). The underlying shape of the design space was consistent for all cases, whereby increased socket press fit resulted in a reduction in the pressure at the residuum tip to zero and an increase in pressure at the tibial tuberosity and fibula head. However, past the threshold press fit where the residuum tip pressure reached zero, the tibial tuberosity and fibula head pressure continued to increase with press fit. Minimising the residuum tip pressure also reduced the distal soft tissue strain. The residuum tip pressure plateaued at a maximum when the press fit was below 1%. Oversizing the socket (i.e. negative press fit) was shown to maximise residuum tip pressure and distal soft tissue strain while minimising longitudinal shear at the posterior calf.

## Discussion

This study presents the first use of a parametric, real-time, FEA-driven model to explore the relationship between residual limb morphology, soft tissue compliance and prosthetic socket design. This allows visualisation of the underlying mechanics between a subset of variables that the prosthetist considers during the patient-specific socket design process. The ability to sweep across the design space enables the variability within this system to be predicted quantitatively, which would not be feasible using experimental techniques on such a scale.

This also demonstrates a meaningful application of SSM applied to transtibial amputated residuum surface shapes to characterise the variation in geometry across a population. The first two PCs used in this study were found to contain only gross limb shape variability which was desired to inform the parametric FE model with a representative population. These PCs have previously been used with linear discriminant analysis as a classification technique between residual limb shapes (Worsley et al. 2015). In contrast to many SSMs which only capture anatomy and sometimes pathology variation (Babalola et al. 2008), the first PC in this model corresponded to a surgical variation of amputation height. The SSM was constructed from scans of rectified casts and thus exhibited non-anatomic socket design features such as the proximal-posterior ‘backslab’ build-up for hamstring relief during knee flexion. These were removed in the present model by using the MRI baseline mesh’s PC scores for all except PCs 1 and 2.

Further, the use of RBFs for mesh morphing enabled simple integration with the SSM model, allowing the tissue nodes to be displaced while the bone was fixed. As this method relied on solving linear equations instead of requiring a PDE solver as in other mesh morphing methods (Bryan et al. 2010), computational efficiency was achieved while preserving mesh quality.

The model’s sensitivity analysis demonstrates the complexity of pressure prediction at the residuum tip, particularly at small press fits. This was supported by regression analysis, where the highest NRMSE was observed at the residuum tip. When the residuum tip pressure was close to or at zero, the surrogate would often predict negative pressure values. This effect is due to the shape of design space where there is a sudden discontinuity at zero, to which the Kriging model attempts to fit a smooth function. Increasing the number of training data points was shown to reduce this effect.

The nonlinearity of the model response was particularly apparent at the residuum tip, whose load bearing ability is a key consideration in socket design (Persson and Liedberg 1982). The surrogate predicts that the magnitude of this load is highly sensitive to both the socket design and morphological variables of the model. This model indicates that shorter residual limbs will result in higher interface pressures as there is less area to distribute the same load. Furthermore, in this case, a tighter fitting socket would be required to off-load the residuum tip. Short limbs are known to be more challenging to fit, and the higher pressures predicted support this (Bowen et al. 2005). Longer, more bulbous limbs were predicted to decrease the pressure over the bony prominences of the residual limb. This is likely due to the increased soft tissue coverage and greater surface loading area contributing to a distribution in the pressure over the limb.

This model also highlighted the interplay between different biomechanical metrics. An increase in longitudinal shear around the main body of the limb was shown to suspend the limb within the socket under load, leading to a reduction in pressure at the residuum tip. This reduction in end bearing also reduced the internal strain around the distal tip of the tibia. Conversely, oversizing the socket (i.e. negative press fit), reduced the bulk shear and increased the tip pressure and soft tissue strain. Further, oversizing the socket caused the tip pressure and soft tissue strain to plateau at a maximum, suggesting the limb had reached a state of near full-end bearing. The effect of an oversized socket is observed clinically, where tissue atrophy results in the residual limb losing volume (Zachariah et al. 2004).

While the soft tissue modulus was shown to have a minor effect on the interface pressure and shear, it was strongly related to the soft tissue strain at the distal tip. Greater magnitudes of relative tibia length (i.e. lower soft tissue coverage over the distal tibia bony prominence) were also shown to increase tissue strain. FE models of amputated lower limbs have been proposed as damage models to predict deep tissue injury based on exposure to strain over time (Portnoy et al. 2009a; Ramasamy et al. 2018). These results demonstrate the importance of accurate characterisation of the soft tissue stiffness, as this parameter will strongly influence any strain-based prediction of injury. Further, as residual limbs become established, they go through an adaptive process and stiffen. To this end, FE models should be used with caution when defining an absolute threshold of injury. A more appropriate application may be on a comparative basis, for example identifying those patients at most risk and evaluating the range of corresponding prosthesis options.

### Limitations

The present study only considered the effect of uniaxial loading to replicate a double-leg stance. The interaction between the residual limb and prosthetic socket is a highly dynamic process (Tang et al. 2015). This has been simplified to contain the dimensionality of the study. However, future studies should incorporate either quasi-static or fully dynamic load cases from gait analysis, and could use surrogate modelling to characterise the effects of loading variability (Galloway et al. 2013) and misalignment (Kobayashi et al. 2013). The bone scaling used in this study was based on linear scaling from the tibial tuberosity; therefore, it does not account for the variation in bone profile across populations. Future studies could use a SSM of the tibia to introduce population-representative variation in bone morphology. The coefficient of friction between the liner and socket was based on the literature data rather than experimental testing, which will affect the shear forces transmitted at the interface. In addition, this study only considered a simplified total surface bearing socket design with the press fit controlled by three points along its length. Future studies may also consider other, more complex parametric models of socket design. Such a model would be able to incorporate the local rectifications that are necessary to reduce pressure over the bony prominences of the residual limb, typically adopted in design principles such as patella tendon bearing sockets. In the present study, these local rectifications were not considered, leading to high pressures over the bony prominences at high levels of global press fit.

Pressure and shear sensors (Laszczak et al. 2015) and lab-based residuum-socket simulators (McGrath et al. 2017) measure the interaction between the residual limb and socket and could be used to reinforce the findings of this study. TSB sockets have been predicted to produce pressure across the limb between 50 and 100 kPa during gait (Dumbleton et al. 2009). This is higher than the simulated pressures in the present study, likely due to the higher forces and moments produced during gait. While it would not be feasible to validate every training data point due to the invented residuum shapes, need to fabricate each socket design and the time taken to run the physical tests, a limited number of studies could be performed to validate some of the underlying mechanisms observed in the model.

### Clinical application

The surrogate model would facilitate automated socket design for an individual lying within the training population using optimisation strategies in a relatively short time; 10,000 surrogate function calls could be evaluated in around 5 min. However, caution should be exercised with such an approach. The selection of an appropriate objective function is challenging, as it requires relating biomechanical outputs such as pressure and shear stresses to clinically relevant metrics such as comfort, stability and highly subjective pain thresholds. Further, during socket fitting, local modifications must be made in case of sensitive regions associated with soft tissue injury or neuroma which are identified during limb assessment but would not be in the computational model. Such an approach would also neglect important psychological aspects during the socket fitting process (Pezzin et al. 2004). This reinforces the importance of a skilled prosthetist within the design of the socket.

Alternative workflows using a FEA solver coupled to a CAD package have previously been proposed (Goh et al. 2005; Colombo et al. 2013). The method presented in this paper, however, overcomes many of the data, software, equipment, computational expense and training barriers associated with performing an FE simulation for each new data point.

To leverage both the skill and experience of the prosthetist with the biomechanical predictions of the model, a PCA-Kriging approach was used for real-time, full-field visualisation of the surrogate. It is anticipated that such a tool could be integrated with an existing CAD socket design software to support the prosthetist. Residuum shape could be matched to the SSM through surface scans, which are already taken in-clinic, bone length through use of planar X-rays, and tissue stiffness using indenters (Petron et al. 2016). The socket design variables would then be selected by the prosthetist within the design process. Such a tool could also enhance user engagement in prosthesis design, which may deliver improved confidence as has been reported anecdotally with CADCAM methods.

## Conclusion

This study’s objective was to develop a surrogate model to allow equivalent predictions to single FEA solutions, across a broad population of amputated residual limb anatomical and surgical variability, and prosthetic socket designs, with sufficiently reduced computational expense for clinical use. The presented framework represents a substantial step towards using quantitative tools to predict the performance of prosthetic socket design prior to manufacture. This study represents the first use of statistically driven morphological variation and parametric prosthetic socket design in predicting the biomechanical response of the residual limb to socket loading. Further, the use of PCA-Kriging to produce a real-time, full-field rendering of the pressure and shear distribution on the residual limb demonstrates a method by which the surrogate could be implemented in a clinical setting. Such a tool would provide the prosthetist with a real-time prediction of socket fit embedded within their CAD package, as part of a more informed socket design process.

## Data Availability

Supporting data are openly available from the University of Southampton repository at http://dx.doi.org/10.5258/SOTON/D0848.
